# Latent profile of personality traits for American older adults and its transition during the COVID-19 pandemic

**DOI:** 10.3389/fpsyt.2024.1358000

**Published:** 2024-10-15

**Authors:** Mingqi Fu, Jing Guo, Hao Kang, Xiaorui Huang

**Affiliations:** ^1^ School of Public Administration, Central South University, Changsha, Hunan, China; ^2^ Department of Health Policy and Management, School of Public Health, Peking University, Beijing, China; ^3^ School of Humanities and Social Science, The Chinese University of Hongkong, Shenzhen, China; ^4^ School of International Relations, Huaqiao University, Xiamen, Fujian, China

**Keywords:** COVID-19 pandemic, frail elderly, latent transition analysis, personality, psychological response

## Abstract

**Background:**

The impact of COVID-19 on older adults’ personality development is essential for emergency management but under-researched. This study seeks to explore the personality profiles of older adults living in the United States and how these profiles transitioned during the pandemic.

**Methods:**

Longitudinal data were collected from 3,550 adults aged 60 and older who participated in both the 2016 and 2020 waves of the Health and Retirement Survey (61.18% female, mean age 65.85 in 2016). Personality traits were assessed using the Midlife Development Inventory. COVID-19-related experiences including pandemic concerns, restricted healthcare access, financial instability, work challenges, disrupted social connections, and mutual aid behaviors. Latent Profile Analysis and Transition Analysis were used for analysis.

**Results:**

Three distinct personality profiles were identified: Well-adjusted, Moderate-adjusted, and Poor-adjusted. About 42% of respondents experienced personality changes during the pandemic. Higher levels of COVID-19 concern were linked to an increased likelihood of transitioning to Poor-adjusted from Moderate (OR=1.06, p<0.05) or Well-adjusted (OR=1.05, p<0.01). Challenges such as healthcare delays and financial hardships hindered transitions from Poor- to Moderate-adjusted (Healthcare delay: OR=0.39, p<0.05; Financial hardships: OR=0.67, p<0.05) but increased the likelihood of Moderate-adjusted individuals transitioning to Poor-adjusted (Healthcare delay: OR=1.46, p<0.05; Financial hardships: OR=1.51, p<0.05). However, Poor-adjusted individuals who provided help to others were more likely to transition to Moderate-adjusted (OR=2.71, p<0.01).

**Conclusions:**

Personality transitions during crisis are significant among older adults. Future interventions should focus on addressing traumatic concerns, encouraging helping behaviors, and mitigating healthcare and financial challenges to support older adults’ personality development during crisis.

## 1 Introduction

Personality traits by definition are descriptions of individuals based on relatively stable patterns of behaviors, thoughts and emotions (1), which may flow into the decision-making process and have significant impacts on various aspects of life (2). For older adults, personality plays a crucial role in executive functioning and the development of mental health issues, such as dementia and depression (3). In current studies, the most widely used system of personality traits is the Five-Factor Model (1), which organizes numerous traits into five broad dimensions: neuroticism, extraversion, agreeableness, openness to experience, and conscientiousness.

These dimensions are considered as distinct and combined to affect how individuals cope with stress (4). For instance, neuroticism, characterized by emotional instability, is linked to maladaptive coping strategies like avoidance and worry, hindering effective stress management (5). In contrast, extraversion, marked by sociability and assertiveness, encourages active problem-solving and optimism (6), while agreeableness, associated with cooperation and compassion, fosters social support and interpersonal effectiveness (5). Openness to experience, which includes creativity and intellectual curiosity, aids in adapting to complex situations. And, conscientiousness, involving determination and planning, is vital for success in structured, goal-oriented environments (7).

Despite their distinctiveness, it is worth noting that these traits can be correlated to some extent. Existing research suggests that individuals high in conscientiousness may also demonstrate agreeable tendencies, as they tend to be reliable and cooperative in their interactions with others (8). This interconnectedness indicates that while the Five-Factor Model provides a framework for understanding distinct traits, individuals can exhibit a combination of these traits that interact and influence each other.

### 1.1 Latent profile of personality traits in individuals’ late life

The study of individuals’ personality traits has traditionally been explored using variable-centered methods, which overlook the interconnected nature of these traits within individuals (9). Alternatively, a person-centered methodology such as latent profile analysis (LPA) can identify person-specific patterns in multiple traits and group individuals into subgroups with similar personality organization (10).

There is no consensus on the number or configuration of personality profiles in older adults. Many studies, based on the vulnerability model, have identified three to four profiles (4). The most common profiles are: Resilient (low neuroticism, average to high levels of other traits), Overcontrolled (low extraversion and emotional stability, high conscientiousness), and Undercontrolled (low emotional stability and conscientiousness, high extraversion) (11, 12). More recent studies suggest a fourth profile, the Vulnerable or Poor-adjusted, characterized by high neuroticism and low levels of the other traits (13, 14). Another proposed four-profile model includes Vulnerable, Moderate (average levels of all traits), Resilient, and Undercontrolled profiles (15). Some studies, such as Van der Wal’s in the Netherlands, identified only two profiles: Resilient and Distressed (16). Additionally, a five-profile model has been noted in various countries, with one predominant Well-adjusted (Resilient) profile and four others with distinct characteristics (17, 18). These profiles’ prevalence varies significantly, with the most wide-known Resilient profile ranging from 10.10% to 61.40% (19). This variability may be due to methodological differences including sample size, age range, instruments used, and analytic techniques (13), as well as cultural and socioeconomic factors (20). Thus, this study focuses on older adults in the U.S. to provide further clarity on personality profiles in late adulthood.

### 1.2 Personality traits changes and life events

Traditional studies often consider personality as a relatively stable characteristic linked to demographic factors like gender, race, age, and socioeconomic factors (21, 22). Recently, there has been a growing focus on understanding personality change as a result of the information-coping process. According to the cognitive-adaptive trait theory, personality development is associated with strategies for adapting to environmental opportunities and pressures, with cognitive skills and self-knowledge acting as mediators (23). Personal events in late life, such as retirement, onset of health issues, and the loss of loved ones, have been examined as factors that drive personality changes (24, 25). In contrast, collective stressful life events such as natural disasters seemed to be nonsignificant (26). However, there is still limited research on how personality changes among older adults during the COVID-19 pandemic.

The COVID-19 pandemic might be a driver for older adults’ personality changes due to the unique challenges that individuals faced during this period. Older individuals, in particular, may experience heightened levels of anxiety and stress stemming from concerns about their susceptibility to the virus and fears surrounding mortality. If these concerns exceed their sense of control, it could lead to dysfunctional aspects of self-regulation, resulting in increased emotional instability (27). Moreover, older adults during the pandemic may encounter more severe deprivations that prompt changes in their personality. Exposure to the coronavirus puts older adults at a greater risk of infection and experiencing more severe disease syndromes. Once infected, older adults may undergo shifts in their worldview and understanding of their place in the world due to impacts of the compromised central nervous system and subsequent negative ruminations (28). Additionally, compared to their younger counterparts, older adults are more likely to face challenges across various aspects of daily life during the pandemic, including accessing healthcare, maintaining financial sustainability, fulfilling daily work responsibilities, and sustaining social connections. For instance, social distancing measures and lockdowns have posed challenges for older adults who are not accustomed to tele-healthcare, making non-infectious healthcare services harder to access (29). Moreover, older individuals may experience increased financial strain as navigating life on a limited budget becomes more challenging, while a significant portion of disadvantaged older adults lack emergency savings (30). The challenges associated with COVID-19 could lead to increased feelings of isolation and insecurity, which may result in withdrawal or a stronger desire for social interaction, ultimately affecting individuals’ personalities during the pandemic (31).

Despite the challenges faced by older adults during the COVID-19 pandemic, those who are motivated and resilient may adopt new coping mechanisms, such as engaging in mutual help behaviors to combat initial challenges. Previous evidence suggests that during emergencies, interpersonal assistance in communication, shopping and other essential activities becomes more prevalent, which may stimulate positive changes in older adults’ personality, such as increased levels of agreeableness and extraversion (32). However, there are also concerns and debates regarding the potential negative effects of giving and receiving help. For older adults, especially those at higher risk during a pandemic, providing assistance to others may lead to caregiver stress, burnout, and strain on their own health, potentially contributing to increased emotional instability (33). Similarly, receiving help may sometimes be perceived as a loss of independence, impacting one’s sense of intelligence and autonomy (34). Furthermore, the dynamics of mutual help can vary based on individuals’ preexisting personality characteristics (35). Some older adults may thrive in caregiving roles, finding fulfillment and purpose in supporting others, while others may experience conflicts related to expectations, boundaries, and reciprocity in social interactions. Thus, it is essential acknowledge the complexity and potential controversies surrounding the dynamics of giving and receiving help when considering older adults’ personality development within the unique context of the COVID-19 pandemic.

### 1.3 Aims and hypothesis

The main goal of this study is to expand upon prior research by examining transitions in personality traits among older adults within a COVID-19 context, which will be achieved by identifying distinct profiles both before and during the pandemic, utilizing a person-centered approach. Additionally, the study will explore how COVID-19 experiences, including concerns, various challenges and mutual help behaviors, may relate to the development of individuals’ personalities.

The first hypothesis is exploratory in nature, positing that there are distinct patterns in the personalities of older adults. The second hypothesis relates to personality transitions among individuals living under the pandemic threats. Drawing from insights in previous studies (27, 31), it is anticipated that older individuals experiencing the pandemic to a greater extent (e.g., higher levels of COVID-19 concern, encountering more COVID-19-related difficulties, and engaging in mutual help behaviors) are more likely to undergo changes in their personality patterns. However, individuals with different personality profiles before the pandemic might exhibit varied trajectories in their personality development in response to COVID-19 experiences. This expectation is based on findings from prior research (35) and highlights the complexity of how individuals’ pre-existing personality traits may interact with pandemic-related experiences to shape their personality changes over time.

## 2 Methods

### 2.1 Research design and participation

Data in this study was obtained from the 2016 and 2020 waves of the Health and Retirement Survey (HRS). HRS is a national longitudinal study focusing on adults aged 45 and older in the United States. Since its inception in 1992, the HRS has been conducted every two years and enrolled over 15,000 individuals per wave (more details see https://hrs.isr.umich.edu). The 2020 wave of the HRS took place from June 2020 to May 2021, a period marked by fluctuations in COVID cases and rapid advancements in scientific understanding, policies, and coping strategies related to the pandemic (36). Significantly, prior to the survey launch, the federal government declared a national emergency in March 2020. Subsequently, lockdown measures and stay-at-home orders were widely implemented by May, leading to frequent delays in healthcare access, financial difficulties, disrupted job opportunities, and interrupted social networking activities among older adults (37). Consequently, the survey included a section to capture individuals’ perceptions and experiences of the pandemic since March 2020. This allowed researchers to analyze the immediate impact of the initial outbreak of the pandemic on the multidimensional living conditions of older adults. For information on ethical approval, sampling design, informed consent, response rates, and survey content of HRS, readers are directed to Fisher & Ryan (38).

In this study, due to the limited participation of older adults in consecutive waves of psychological assessment, we used personality data from 2016 as a proxy for participants’ pre-pandemic status. The initial sample size was 4,101. Following the World Health Organization’s definition of individuals aged 60 and above as older adults, who were identified as particularly vulnerable during the COVID-19 pandemic (39), we set the age limit at 60 years. After excluding individuals below this age (342 observations) and those with missing information on two or more of the COVID-19 experiences (124 observations) or either wave of personality trait assessments (85 individuals), our final analysis included 3,550 cases. Missing data were handled with Listwise deletion.

### 2.2 Measures

Personality in this study was assessed using the Midlife Development Inventory (MIDI), an inventory that is commonly employed in large panel surveys of adults (40). The MIDI comprises a total of 26 adjectives describing individuals’ big five traits, with specific items allocated for extraversion, agreeableness, conscientiousness, neuroticism, and openness. Respondents are asked to rate how well each item describes them based on their feelings at the time of the interview, using a scoring system ranging from 1 (not at all) to 4 (a lot). The average score across the included items was calculated for each trait, with higher scores indicating more pronounced sub-scaled traits. The MIDI has been validated as a reliable tool for assessing personality traits in older adults (41). The overall Cronbach’s alpha coefficient for the MIDI was 0.76 in the 2016 wave and 0.77 in the 2020 wave, indicating good internal consistency. Through latent profile analysis, personality was then categorized into three profiles: Well-adjusted, Moderate-adjusted, and Poor-adjusted.

COVID-19-related experiences among older adults included concerns about the pandemic, deprivations resulting from pandemic-related measures, and mutual help behaviors since March 2020.

COVID-19 concern was evaluated using a 10-point scale, asking respondents to rate “how concerned are you about the pandemic.” Higher scores indicated greater levels of COVID-19 concerns.

COVID-19-related difficulties encompassed various challenges that individuals had since March 2020. These difficulties included: 1. Being infected, which assessed the risk of exposure to the virus with the question “Have you ever been diagnosed with COVID-19?” 2. Healthcare delays, measured through five questions about their experiences such as delayed doctor visit. 3. Financial hardships, evaluated with six items like “could not pay medical bills due to COVID reasons.” 4. Work challenges, consisting of five items such as “I lost my job because of the pandemic.” 5. Impeded social networking, indicated by five types of experiences like “had to cancel family gatherings due to COVID restrictions.” During the survey, participants reported whether they had experienced each item during the pandemic, with a score of 1 indicating “yes” and 0 indicating “no”. Scores were then summed to reflect the level of corresponding difficulties, with total scores of 5 for healthcare delay, 6 for financial hardships, 5 for work challenges, and 5 for social connection difficulties.

Mutual help behaviors against the pandemic were assessed as received help and gave help. For received help, participants were asked if anyone outside their household helped them with bills or chores during the pandemic, with a response of 1 for “yes” and 0 for “no”. Gave help was measured by asking if participants helped anyone outside their household with bills or chores during the pandemic, with a score of 1 for affirmative responses and 0 otherwise.

Covariates in this study included age, gender (male/female), marital status (married or partnered/single), race (white/black/others) and Medicaid eligibility (yes/no). These variables have been examined to associated with older adults’ personalities (21, 22).

### 2.3 Analytic strategies

Descriptive analyses were conducted for all variables as of 2016 and 2020, respectively. For continuous variables, means and standard deviations were reported, while for categorical variables, numbers and percentages were reported.

To examine the development of older adults’ personalities during the pandemic and the influence of COVID-19 experiences on the transition probability, we used the Bolck-Croon-Hagenaars method (BCH) Latent transition analysis (LTA). The BCH-LTA involves three steps. First, by conducting LPA at each time point, we determined the optimal number of latent profiles for each time. Second, we independently estimated and assigned BCH weights to each sample, solely relying on the latent class indicators specific to that particular time point. Third, we estimated the LTA model with covariates.

In the first phase, we conducted LPA to identify unobserved clusters of individuals sharing similar personality profiles. This analysis was performed separately for the years 2016 and 2020 to determine if the combination of personality indicators remained stable over time. The LPA process involved estimating models with varying numbers of profiles, starting with a one-profile model and progressing to more complex models with increasing numbers of profiles. Each model (K profiles) was compared to the previous model with one less profile (K-1 profiles) until the larger model (K profiles) was not found to be significantly better than the one with fewer profiles. Various indicators were used to evaluate the models, including Akaike Information Criterion (AIC), Bayesian Information Criterion (BIC), sample size-adjusted BIC (ssaBIC), entropy, values of the Lo-Mendell-Rubin Test (LMRT), and the Bootstrap Likelihood Ratio Test (BLRT). Generally, a more optimal model is indicated by lower AIC, BIC, and ssaBIC values; relatively higher entropy (> 0.60 is considered acceptable); the smallest class size exceeding 5%; and significant LMRT and BLRT values (42). Last, membership for individuals in each profile was determined based on their average posterior probabilities.

Then, the BCH weights were calculated and employed to compare differences between profiles in terms of related demographic and socioeconomic characteristics. Unlike methods that assume no error, BCH considers uncertainties in classification. This approach saves the posterior probabilities and model class assignments from the optimal classification model. Next, the inverse logits of individual-level classification error rates are computed and then used as profile weights in estimating distal variables, such as profile characteristics. More information about BCH methods can be found in Nylund-Gibson’s previous work (43). In the current study, pairwise comparisons of the characteristics between profiles were conducted simultaneously using Wald’s test.

In the third stage, we utilized latent transition analysis (LTA) without covariates, a type of autoregressive model, to model changes in group membership over time (44). Transition probabilities are conditional probabilities that describe the probability of being in a given state, conditional on the state from the previous time point. For example, the probability of individual i transitioning into a state m (m=1, …, s) from state k (k=1, …, s) could be estimated using the following equation:


$$\tau_{ikm}=\frac{exp\left(\alpha_{m}+\beta_{km}\right)}{\Sigma_{s=1}^{s}exp\left(\alpha_{s}+\beta_{ks}\right)}$$

Where the term in the denominator for the reference class (last class) is 1 because $\alpha_{s}$ =0 and $\beta_{ks}$ =0 for standardization (45). Older adults who did not experience a change in personality traits from 2016 to 2020 were categorized as “stayers”, while those who did were categorized as “movers”.

Finally, the COVID-19 experiences variables were added as covariates to the mixture model following a BCH-LTA approach (43). The effect of the covariates on the transition probability odds ratios were reported.

All tests were two-sided with an alpha level of 0.05 for statistical significance and the analyses were carried out using Mplus version 8.

## 3 Results

### 3.1 Descriptive analysis

Among the older adults in the sample, the majority were female (N=2171, 61.18%) and identified as non-Hispanic White (2599, 73.48%). The average age at baseline was approximately 65.85 years (Standard deviation, SD=10.16), with around 34.01% (N=1207) of participants reporting being single. By the year 2020, the percentage of single individuals had increased to 38.77% (N=2168), and the number of Medicaid recipients rose from 9.56% (N=338) in 2016 to 10.55% (N=371). During the pandemic, older adults expressed relatively high levels of COVID-19 concern (Mean, M=7.77, SD=2.59). Although only 5.06% (N=127) of respondents had been diagnosed with the disease, they experienced notable challenges in social networking (M=1.99, SD=1.54) and financial hardships (M=1.09, SD=0.55). Approximately 23% (N=821) of individuals received social support to cope with the pandemic, and about 36.51% (N=1296) reported having helped others.

In terms of personality traits, there was an increase in the mean score for neuroticism from 1.93 (SD=0.36) in 2016 to 1.95 (SD=0.36) in 2020, indicating a heightened level of emotional instability and irritability among older adults during the COVID-19 threats. Conversely, extraversion decreased from 3.21 (SD=0.36) in 2016 to 3.15 (SD=0.34) in 2020; openness decreased from 2.95 (SD=0.33) to 2.93 (SD=0.34); agreeableness declined from 3.53 (SD=0.24) to 3.49 (SD=0.27); and conscientiousness declined from 3.40 (SD=0.23) to 3.36 (SD=0.25) in 2020. Further details are provided in **Table 1**.

**Table 1:** Mean and standard deviation for socioeconomic characteristics, pandemic-related experiences and personality traits variables for the wave 2016 and wave 2020 (N=3550).

		Wave-2016	Wave-2020
		*M*/N (*SD*/P)	*M*/N (*SD*/P)
**Gender**	Male	1378 (38.83)	
	Female	2172 (61.18)	
**Race**	White	2599 (73.48)	
	Black	640 (18.09)	
	Others	298 (8.43)	
**Age**		65.85 (10.16)	69.84 (10.24)
**Marital Status**	Married/Partnered	2342 (65.99)	1373 (61.23)
	Single	1207 (34.01)	2168 (38.77)
**Medicaid Eligibility**	Yes	338 (9.56)	371 (10.55)
	No	3198 (90.44)	3147 (89.45)
**COVID-19 Concern**			7.77 (2.59)
**COVID-19-related difficulties**	Being infected		127 (5.06)
	Healthcare delay		0.496 (0.896)
	Work challenge		0.213 (0.578)
	Financial hardships		1.091 (0.547)
	Impeded social networking		1.992 (1.536)
**Mutual Help Behaviors** **against COVID-19**	Received support		821 (23.13)
	Gave support		1296 (36.51)
**Personality Traits**	Neuroticism	1.93 (0.36)	1.95 (0.36)
	Extroversion	3.21 (0.32)	3.15 (0.34)
	Openness to experience	2.95 (0.33)	2.97 (0.34)
	Agreeableness	3.53 (0.24)	3.49 (0.27)
	Conscientious	3.40 (0.23)	3.36 (0.25)

M, Mean; N, Counts; SD, Standard Deviation; P, Proportion.

Mean and Standard Deviation were reported for continuous variables, and Counts and Proportion were reported for categorical variables.

### 3.2 Exploration of latent personality profiles for wave 2016 and wave 2020

**Figure 1:**
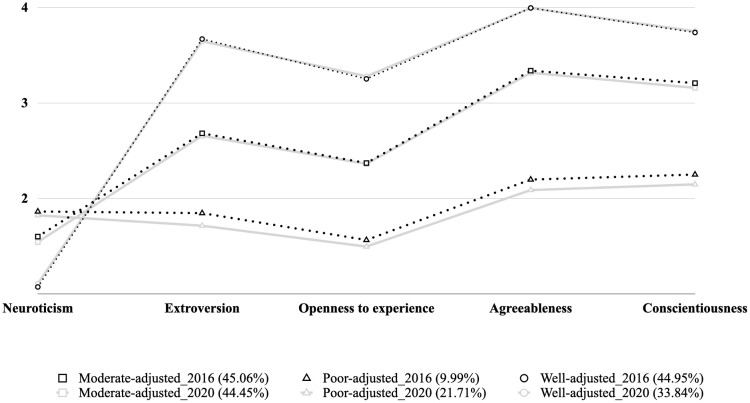
Conditional response means of the 3-class solution.

The exploration of measurement model helps to identify whether similar classes emerge at each time point of interest. As shown in **Table 2**, both in wave 2016 and wave 2020 the three-class solution fitted the data best. Based on the estimated personality profile of respondents from each latent class (See **Figure 1**), we further summarized the detected profiles as Poor-adjusted, Moderate-adjusted, and Well-adjusted.

**Table 2:** Model fit indices.

	Solution	Loglikelihood	AIC	BIC	ssaBIC	Entropy	LMR		BLRT		Class Proportion			
							2LL	P	2LL	P				
**Wave-2016**	1-class	-14236.74	28456.26	28555.22	28523.44									
	2-classes	-12510.42	25036.63	25151.64	25100.80	0.79	3452.62	0.000	3452.63	0.000	32.10	67.90		
	**3-classes**	**-12034.94**	**24135.36**	**24249.72**	**24179.82**	**0.79**	**950.96**	**0.006**	**950.96**	**0.000**	**45.06**	**9.99**	**44.95**	
	4-classes	-11802.84	23765.55	23834.57	23745.60	0.76	464.20	0.120	464.20	0.000	3.00	25.60	49.50	21.90
**Wave-2020**	1-class	-14708.65	29326.52	29499.04	29467.26									
	2-classes	-12935.69	25863.24	26002.19	25951.35	0.78	3545.89	0.000	3475.05	0.000	32.80	67.20		
	**3-classes**	**-12356.13**	**24756.26**	**24892.10**	**24822.20**	**0.79**	**1159.13**	**0.000**	**1135.98**	**0.000**	**44.45**	**21.71**	**33.84**	
	4-classes	-12152.33	24501.24	24533.56	24444.59	0.76	407.59	0.012	399.45	0.013	4.20	26.30	25.00	44.50

AIC, Akaike Information Criterion; BIC, Bayesian Information Criterion; ssaBIC, sample size-adjusted BIC; LMRT, Lo-Mendell-Rubin Test; BLRT, Bootstrap Likelihood Ratio Test. The bold values indicate that the 3-classes solutions have the best model fit indices.

The Poor-adjusted profile was characterized by a relatively high level of neuroticism and low levels of the other four traits. Individuals in the Poor-adjusted profile might exhibit heightened emotional reactivity to stressors but may be less capable of coping with them effectively. On the other hand, the Well-adjusted profile (or Resilient) was the opposite of the Poor-adjusted profile, with individuals believed to experience adaptive outcomes in the face of stressful circumstances. The Moderate-adjusted profile possessed a middle level of all traits, representing a resilience profile but possibly with a slightly lower level of adaptation compared to the Well-adjusted profile. The proportion of individuals in the Poor-, Moderate-, and Well-adjusted groups was 9.99%, 45.06%, and 44.95% in 2016, respectively, and 21.71%, 44.45%, and 33.84% in 2020.

**Supplementary Table S1** further examined the sociodemographic characteristics associated with older adults’ personality profiles. The results indicated that all profiles significantly differed from one another in terms of gender and Medicaid eligibility, both before and during the pandemic. The Poor-adjusted profile tended to have fewer females (44.74% in 2016; 50% in 2020) and more individuals receiving Medicaid (15.86% in 2016, 19.83% in 2020) compared to other profiles. In a pandemic context, individuals of White race seemed more likely to be in the Moderate-adjusted (75.11%) or Well-adjusted (72.76%) profiles rather than the Poor-adjusted profile (66.12%).

### 3.3 Exploration of latent transition patterns and its relationships with COVID-19 experiences

**Table 3** illustrated personality changes between 2016 to 2020 using cross-sectional LPAs. The values along the diagonal describe stability in personality, while the off-diagonals reflect movements. As observed, 64.30% of individuals with a Moderate-adjusted personality, 49.9% of those in the Poor-adjusted group, and 54.3% of the Well-adjusted group would remain in the same group during the pandemic.

**Table 3:** Preliminary transition tables based on trait classifications from the cross-sectional LPA modeling results.

	Moderate-adjusted-2020	Poor-adjusted-2020	Well-adjusted-2020
**Moderate-adjusted-2016**	0.64	0.20	0.16
**Poor-adjusted-2016**	0.29	0.50	0.21
**Well-adjusted-2016**	0.28	0.18	0.54

The detailed stayer-mover patterns are presented in **Table 4**. Overall, approximately 58.39% of older adults maintained stable personalities throughout the pandemic, while nearly 41.61% experienced some degree of personality changes. The most common patterns of movement were from Well-adjusted individuals to Moderate-adjusted (12.58%) or Poor-adjusted (7.95%), and from Moderate-adjusted to Poor-adjusted (8.77%). These changes are viewed as negative, as individuals may find it challenging to engage in adaptive behaviors once these transitions occur. However, there were also some positive changes observed. Around 7.3% of the sampled individuals shifted from Moderate-adjusted to Well-adjusted, and to a lesser extent from Poor-adjusted to Moderate-adjusted (2.89%) or Well-adjusted (2.11%).

**Table 4:** Percent of older adults in each pattern of personality transition, orders by the frequency of the patterns, by movers then stayers.

	Wave-2016	Wave-2020	Frequency
**Movers (41.61%)**	Well-adjusted	Moderate-adjusted	12.58%
	Moderate-adjusted	Poor-adjusted	8.77%
	Well-adjusted	Poor-adjusted	7.95%
	Moderate-adjusted	Well-adjusted	7.30%
	Poor-adjusted	Moderate-adjusted	2.89%
	Poor-adjusted	Well-adjusted	2.11%
**Stayers (58.39%)**	Moderate-adjusted	Moderate-adjusted	28.98%
	Well-adjusted	Well-adjusted	24.42%
	Poor-adjusted	Poor-adjusted	4.99%

**Table 5** explores whether COVID-19-related experiences are related to transition probabilities. Notably, a higher level of COVID-19 concern was associated with a lower chance for Poor-adjusted individuals to transition into the Moderate-adjusted (OR=0.95, p<0.001) or Well-adjusted (OR=0.94, p<0.05) personality profiles. Individuals with Moderate-adjusted (OR=1.06, p<0.05) or Well-adjusted (OR=1.05, p<0.01) personalities who experienced heightened COVID-19 concerns were more likely to transition into a Poor-adjusted personality rather than maintaining their previous profiles. However, disease infection did not show a significant relationship with personality changes. Additionally, in the context of COVID-19-related challenges and mutual support behaviors, notable correlations were observed among individuals classified as Poor-adjusted or Moderate-adjusted before the pandemic. Factors such as healthcare delays and financial strain were linked to an increased likelihood of developing a Poor-adjusted personality during the pandemic. Conversely, engaging in helping behaviors towards others appeared to be associated with a higher probability of developing a Moderate-adjusted personality for Poor-adjusted individuals amidst the COVID-19 circumstances (OR=2.08, p<0.01).

**Table 5:** The odds ratio of latent transition probabilities as a function of COVID-19 related experiences (stayers as reference).

	Poor-adjusted		Moderate-adjusted		Well-adjusted	
	P-M	P-W	M-P	M-W	W-B	W-M
**COVID-19 Concern**	0.95***	0.94*	1.06*	0.99	1.05**	1.05**
**COVID-19 Infection**	1.35	0.93	1.37	1.31	1.23	1.19
**Healthcare Delay**	0.39*	0.55	1.46*	0.91	1.01	0.99
**Work Challenge**	0.70	0.83	1.17	0.98	1.16	1.19
**Financial Hardship**	0.67*	0.73	1.51*	0.64	1.19	1.03
**Impeded Social networking**	0.93	0.94	1.02	0.95	0.99	1.12
**Received Support**	0.70	0.30	1.43	0.94	1.08	0.83
**Gave Support**	2.08**	2.48	0.80***	1.44	0.88	1.35

P, Poor-adjusted; W, Well-adjusted; M, Moderate-adjusted, *** P<0.001, ** P<0.01, * P<0.05.

## 4 Discussion

This study with a sample of 3,550 older adults from the United States observed three latent personality profiles: Well-adjusted, Moderate-adjusted and Poor-adjusted. It is noteworthy that during the COVID-19 pandemic, there was roughly a 42% probability of personality transition among older adults. Among these transitions, the most common were Well-adjusted individuals transitioning to the Moderate-adjusted profile (12.58%), followed by transitions from Moderate- to Poor-adjusted (8.77%), and from Well- to Poor-adjusted (7.95%). Moreover, we provided empirical support for the cognitive-adaptation traits theory, showing that heightened pandemic concern and difficulties related to COVID-19 may associated with an increased likelihood of transitioning to the Poor-adjusted profile. Conversely, engaging in supportive behaviors toward others during public emergencies was linked to a higher probability of transitioning from Poor- to Moderate-adjusted.

Several findings of this study warrant further discussion. First, this study identified three distinct latent personality profiles within the sampled older adult population, noting that the majority fell into the categories of Well- or Moderate-adjusted. An interesting observation was the configuration of personality profiles, particularly the prevalence of a middle level of personality expressions among elderly individuals, which was less frequently observed in younger age groups (11–14). This aligns with the notion that personality traits can adapt across the lifespan, with individuals generally becoming more “average” as they age (46). Notably, consistent with previous research (20), neuroticism emerged as the most crucial trait distinguishing these personality profiles, with significant variance observed in over 30% of the independent sample. In terms of profile size, the proportion of Moderate-adjusted and Well-adjusted older adults during the pandemic exceeded 50%, which was notably higher compared to estimates for adults at earlier life stages (47, 48). This aligned with prior findings that younger adults experienced increased neuroticism and decreased agreeableness and conscientiousness than older counterparts during the pandemic (27). It is possible that older individuals, despite facing disproportionate stressors during the pandemic, might possess greater psychological resources such as accumulated life experiences and a broader perspective on life. With which, older adults may view challenges as temporary and part of a larger context, and maintain adaptability during difficult times (49). Moreover, older adults may employ more sophisticated coping strategies to navigate environmental uncertainties. As previous research noted, individuals improve at regulating emotions as they age, with many older adults prioritizing what matters most, focusing on positive aspects of life, and managing stress effectively (50). Thus, this study highlights the potential for positive personality outcomes among older adults facing pandemic threats. Moreover, encouraging the preservation of their psychological resources, fostering adaptive coping strategies, and addressing heightened neuroticism during such challenging times might be crucial.

In addition, we found that males and Medicaid recipients were more prone to being Poor-adjusted, whereas in a COVID-19 context in particular, individuals from non-white racial groups showed a higher likelihood of exhibiting a Poor-adjusted personality. Inconsistent with previous research (21), evidence from this study suggested that older females may have better self-regulation skills based on their biological and social characteristics, leading to a higher likelihood of being Well-adjusted compared to males (51). Additionally, we revealed that individuals enrolled in Medicaid programs were more likely to exhibit Moderate-adjusted or Poor-adjusted personality profiles, which underscored the potential exacerbation of socioeconomic vulnerability among this group. With regard to the significant racial variations in personality profiles, reasons could be attributed to heightened socioeconomic challenges such as lower income and limited access to healthcare among disadvantaged racial groups (52). Therefore, it is recommended that special attention should be given to the vulnerability of males, Medicaid recipients, and individuals from non-white racial groups in terms of personality development during health crises like the COVID-19 pandemic.

Moreover, this study observed frequent changes in the personalities of older individuals during the pandemic, with higher levels of COVID-19 concern significantly linked to negative changes, such as transitioning from a state of Well-adjusted or Moderate-adjusted to becoming Poor-adjusted. This aligned with the Cognitive-adaptive Trait Theory’s assertion that enduring and severe stressors like the COVID-19 pandemic could impact older adults’ personality traits (23). More detailed, older adults experiencing heightened concern about the pandemic might be more vulnerable to enduring distress characterized by fear, insecurity, and uncertainty (27). As a result, this vulnerability increases the likelihood of experiencing heightened emotional instability and reduced levels of extroversion, openness, agreeableness, and conscientiousness. However, contrary to prior research (28), this study did not find a significant link between disease infection and personality changes among older adults. This may be due to older individuals’ tendencies to underestimate and lack awareness of disease infection (53), as well as their inclination to attribute the causes and consequences of infection to external factors (49). Moreover, the short-term nature of the threats posed by contracting the coronavirus may also contributed to the lack of significant personality changes. The clear and simple solutions to these threats might hinder the accommodation of the enduring and dynamic nature of personality development (54). Thus, it might be important to address and support the ongoing concerns of older adults during health crises like the COVID-19 pandemic to foster more adaptive personality outcomes.

Besides, the impact of healthcare delays, financial hardships, and mutual help behaviors on the personality development of older adults was found to vary significantly based on individuals’ pre-existing personality profiles. This study introduced a novel finding that Well-adjusted individuals, who might possess more effective coping and reflective strategies, experienced less impact on their personality traits due to pandemic-related difficulties. In general, Well-adjusted older adults may have a greater reserve of resources, leading to a reduced impact of COVID-19-related challenges such as income reduction and healthcare delays on their personality development (55). Also, it appears that Well-adjusted individuals may have a higher threshold for negative personality changes, possibly due to their ability to maintain stable personalities through positive social comparisons and lower negative self-reflection compared to their peers (56). Conversely, for Poor-adjusted and Moderate-adjusted individuals, challenges related to healthcare access and financial stability—two critical aspects significantly affected during the pandemic—might disrupt their self-knowledge foundation (57). They were more prone to withdrawing from social connections, engaging in maladaptive ruminations, and adopting passive self-regulation strategies, all of which contribute to negative personality changes (28). Based on these findings, this study proposed that fostering a well-adjusted personality among older individuals might better prepare them for future emergencies. Also, timely intervention and support should be provided to Poor-adjusted and Moderate-adjusted individuals experiencing healthcare delays and financial hardships to mitigate the negative impact on their personality development.

Last, it is noteworthy that the pandemic might also presents opportunities for positive personality development. We added to existing knowledge by demonstrating that offering assistance to others may facilitate the development or maintenance of a Moderate-adjusted personality. Perhaps, engaging in acts of service may shift the focus of older adults from personal worries to the needs of others, potentially reducing self-centeredness and alleviating anxiety or fears (58). Moreover, helping others might provide a sense of purpose and foster social connections, leading to increased self-esteem, reduced feelings of loneliness and isolation, and contributing to emotional stability, extraversion, and openness during difficult times (32). However, contrary to our initial hypothesis, receiving help from others did not show a significant relationship with changes in older adults’ personalities. This could be due to a counterbalancing effect, where increased appreciation for life may be offset by a perceived decrease in self-efficacy through peer comparison (34). Therefore, it might be useful to promote helping behaviors among older adults during emergencies. Individuals with Poor- or Moderate-adjusted personality profiles should be particularly prioritized in these efforts to support their well-being and foster positive personality development.

### 4.1 Strength and limitations

This study is among the first to use a person-centered methodology to explore personality transitions among older adults, considering impacts of the COVID-19 pandemic. The findings provided valuable insights into how older adults’ personality traits may evolve in response to significant stressors like the pandemic, which might inform interventions and support systems aimed at promoting resilience and well-being among this population during challenging times.

However, several limitations should be acknowledged. Firstly, the assessment of pre-pandemic personality used data from four years before the pandemic, potentially introducing confounding factors unrelated to the pandemic that could influence observed personality transitions. Additionally, the impact of the pandemic on personality profiles was assessed only in the first year after the outbreak, highlighting the need for future research to explore if these changes persist in post-pandemic phases. Furthermore, although it appeared reasonable in the temporal sequence to assess the impact of COVID-19 experiences on personality changes during the pandemic, it is important to note that these variables were assessed in a cross-sectional study. Variables like COVID-19 concern, which were estimated using a single self-reported item, should be interpreted cautiously regarding their influence on personality transitions. Therefore, longitudinal evidence with more comprehensive measures is critical for future studies to validate this relationship. Lastly, although cognitive skills and self-knowledge are believed to influence personality changes in older adults, these variables were not directly measured in our study. Additionally, confounders such as co-morbidities may have influenced personality development but were not a primary focus due to data limitations. Future research is needed to empirically investigate how these factors contribute to personality changes among older adults.

## 5 Conclusion

With a sample of 3,550 older adults from the United States, we observed three distinct personality profiles: Well-adjusted, Moderate-adjusted and Poor-adjusted. We found that over 40% of the individuals may experience personality transitions during the pandemic. While a higher level of COVID-19 concern and greater pandemic-related difficulties associated with a higher probability of being Poor-adjusted during the pandemic, engaging in helping services might facilitate changes from Poor-adjusted to Moderate-adjusted. The study extends existing findings and provides novel evidence on the relationship between traumatic experiences and changes in older adults’ personality traits. Identification of personality profiles and its developmental trajectories might be useful in targeting those who may be at risk for undesirable personality changes. To the extent that personality traits are modifiable, the present findings may also provide valuable information for tailoring interventions based on constellations of individual differences in personality, especially for those who are experiencing negative personality changes.

## Data Availability

The original contributions presented in the study are included in the article/**Supplementary Material**. Further inquiries can be directed to the corresponding author.
