# Supplementary material for: Latent profile of personality traits for American older adults and its transition during the COVID-19 pandemic
Source: Front Psychiatry. 2024 Oct 15;15:1358000. doi: 10.3389/fpsyt.2024.1358000 (PMC11522981; doi:10.3389/fpsyt.2024.1358000)
Supplement: Supplementary file 1 [file Table1.docx]

Table S1. Socioeconomic characteristics of older adults from different class of personalities using cross-sectional LPA results: BCH Methods

| **Year-2016** |  | Poor-adjusted | Moderate-adjusted | Well-adjusted |
| --- | --- | --- | --- | --- |
|  | Age | 65.29 a | 65.76 a | 65.98 a |
|  | Gender (% female) | 44.74 a | 57.58 b | 65.78 c |
|  | Marital Status (% Married/Partnered) | 69.30 a | 66.67 a | 65.10 a |
|  | Medicaid eligibility (% Yes) | 15.86 a | 11.79 b | 7.17 c |
|  | Race (% White) | 63.44 a | 75.77 b | 73.00 b |
| **Year-2020** |  | Poor-adjusted | Moderate-adjusted | Well-adjusted |
|  | Age | 69.18 a | 70.38 a | 71.17 a |
|  | Gender (% female) | 50.00 a | 57.32 b | 66.15 c |
|  | Marital Status (% Married/Partnered) | 57.38 a | 62.21 a | 60.89 a |
|  | Medicaid eligibility (% Yes) | 19.83 a | 10.90 b | 8.85 c |
|  | Race (% White) | 66.12 a | 75.11 b | 72.76 c |

Notes: Across rows, values sharing the same subscript indicate nonsignificant differences among profiles.
